# Electrostimulation waveform optimization for enhancing biomass and macromolecule production in *Chlorella vulgaris*

**DOI:** 10.1007/s00449-026-03355-1

**Published:** 2026-06-10

**Authors:** Yaşar Aluç, Osman Kök, Mustafa Doğan, İlhami Tüzün

**Affiliations:** 1 Scientific and Technological Research Application and Research Center, Kırıkkale University, Kırıkkale, Türkiye; 2 Department of Crop and Animal Production, Digital Agricultural Technologies Programme, Delice Vocational School, Kırıkkale, Türkiye; 3 Department of Biology, Kırıkkale University Graduate School of Natural and Applied Sciences, Kırıkkale, Türkiye; 4 Department of Electronics and Automation, Biomedical Device Technology Program, Hacilar Huseyin Aytemiz Vocational School, Kırıkkale University, Kırıkkale, Türkiye; 5 Department of Biology, Faculty of Arts and Sciences, Kırıkkale University, Kırıkkale, Türkiye

**Keywords:** *C. vulgaris*, Alternating current, Carotenoid, Microalga growth, Chlorophyll fluorescence

## Abstract

While electro-technological studies on algae typically focus on direct current, electric field, and high-voltage electric field applications, this study investigates the effects of various alternating current electric waveforms on the biomass production, macromolecular, and carotenoid composition of the microalgae species *Chlorella vulgaris*. The findings reveal that the applied waveforms significantly enhanced the growth performance of *C. vulgaris* compared to the control group. OD680 values increased by 40% in the Pulse-10 group, 33% in the Pulse-90 group, 26% in the Sine, 24% in the Square group and group 19% in the Triangle group. Chlorophyll-*a* content also increased similarly, with the highest increase of 54% observed in the Pulse-10 group. Furthermore, macromolecule and total carotenoid production was supported and increased compared to the control group. In conclusion, these findings contribute to innovative electro-technological applications that enhance the commercial potential of *C. vulgaris* for use in fields such as food, feed, cosmetics, and biodiesel production.

**Supplementary Information:**

The online version contains supplementary material available at 10.1007/s00449-026-03355-1.

## Introduction

Recognized as among Earth’s earliest life forms [9], microalgae possess simple photosynthetic cellular structures and a remarkable ability to rapidly proliferate, allowing them to inhabit diverse environments, including those with extreme conditions [10, 39]. By synthesizing valuable organic compounds such as carbohydrates, lipids, proteins, carotenoids, alkaloids, and fatty acids [4, 19], microalgae find extensive application in sectors like food, cosmetics, and health due to these beneficial biocompounds [5, 52]. Moreover, microalgae are emerging as a significant sustainable, renewable, and environmentally friendly alternative energy source [1]. Accordingly, significant research has been conducted focusing on increasing microalgal biomass and applying innovative technologies for microalgal metabolites [63, 11, 35]. Notably, mathematical and artificial intelligence models have been developed to characterize microalgal biomass production, assessing the impact of factors such as light, temperature, and nutrient consumption [18, 38].

Current research on microalgal biomass and metabolites includes a new approach classified as electro-technological studies, encompassing electric field applications, with Pulsed Electric Field (PEF) technology applications being predominant enhancing microalgal biomass, and extracting lipids or pigments [11, 12, 22, 24, 28, 29, 33, 34, 46]. In their study, Pereira et al. [46] reported that treatment of wet *Chlorella zofingiensis* biomass with a pulsed electric field resulted in an increase of approximately three-to-fourfold the extraction of carotenoids and chlorophyll compared to the control group. Similarly Haberkorn et al., [14] reported that pulsed electric field application during the early exponential growth phase of *Chlorella vulgaris* significantly increased biomass yield by up to 18%, Furthermore Buchmann et al., [3] reported that exposing Arthrospira platensis to a pulsed electric field (PEF) before cultivation resulted in a 13% increase in microalgal biomass. Treatment of microalgae with PEF has been shown to lead to an increase in valuable biocompounds such as lipids, proteins, carbohydrates, and carotenoids [29]. A study by [6] reported that treatment of *Chlorella vulgaris* biomass with 25 kV cm⁻¹ PEF resulted in a 27% increase in lipids compared to the control [6].

In contrast, this study investigates the effect of alternating current (AC) and its applications representing such an electro-technological approach on the biomass enhancement and carotenoid composition of *C. vulgaris*. Despite extensive research on various alternating current (AC) waveforms in other fields [30, 58], AC applications in microalgae have not been sufficiently investigated. The aim of the present study is to evaluate the effect of various AC waveforms on the biomass yield and carotenoid profile of *Chlorella vulgaris*. Distinguishing this work from prevalent research which predominantly focuses on direct current (DC) stimulation, static electric fields, and high-voltage electric field (HVEF) treatments-our methodology involves the direct application of low-voltage AC signals to the culture medium. This approach allows for a precise investigation of how waveform geometry and periodic modulation affect microalgal metabolic pathways.

## Methods and materials

### Strains and culture conditions

*Chlorella vulgaris* microalgae are continuously cultivated in the microalgae culture collection at the Environmental Laboratory of the Department of Biology, Faculty of Engineering and Natural Sciences, Kırıkkale University. Under laboratory conditions, the microalgae were cultivated in Tris-Acetate-Phosphate (TAP) liquid medium containing macronutrients and micronutrients in optimal proportions, at a temperature of 25 ± 1 °C, with a 16:8 h light-dark cycle and a light intensity of 4000 lx (cool white fluorescent light) [16, 27, 57–59]. This strain was initially isolated from the Kapulukaya Dam Reservoir environment, and its identification involved morphological and molecular characterization, including imaging with an inverted microscope and Scanning Electron Microscopy (SEM). Based on the results from the molecular analysis of two gene regions (18 S rRNA, ITS, rbcl, and 16 S molecular markers), it was further confirmed as *C. vulgaris*.

### Distinct electric treatment

**Fig. 1:**
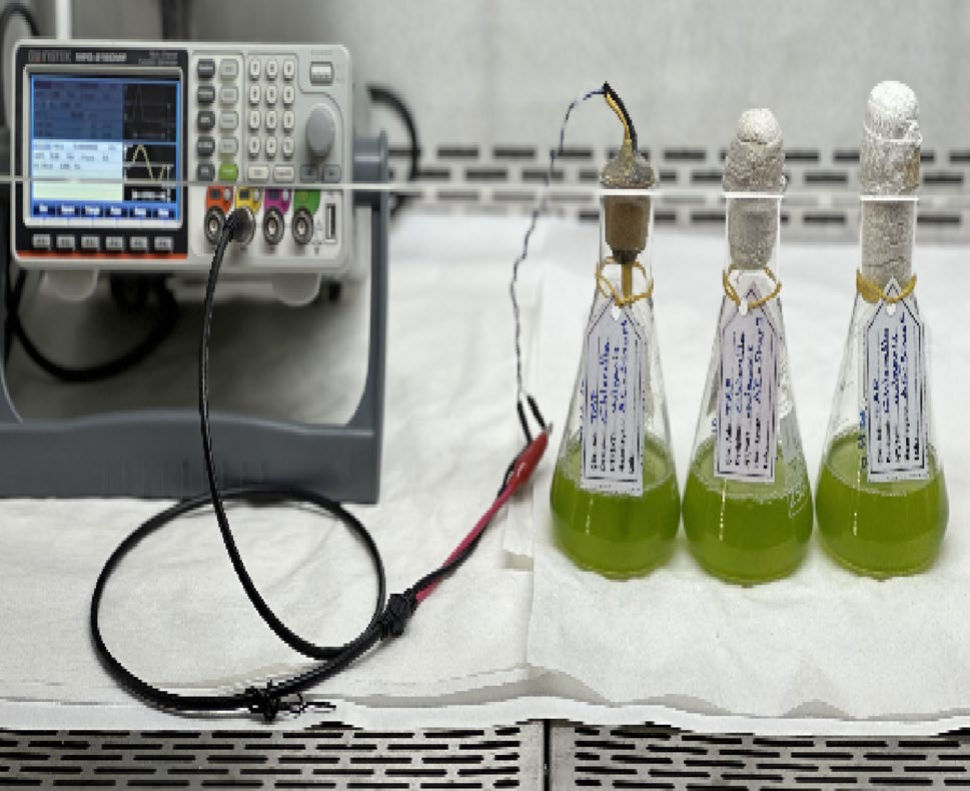
Application of alternating current to C. *vulgaris* culture medium

During cultivation, five distinct electrical signal waveforms sine, pulse (Pulse-90 and Pulse-10), square, and triangular were applied to each culture using an MFG-2000 Series Multi-Channel Function Generator. Titanium metal (non-corrosive) anode and cathode rods spaced 3 cm apart were connected to the signal generator. These electrodes were submerged approximately 1 cm above the bottom of the liquid algal culture medium for 60 s daily, directly applying the distinct electrical waveforms to each culture in triplicate (Fig. 1).

### Measurement of cell pigment concentrations

Starting from the inoculation day, 2 mL samples were collected daily from each culture and placed into eppendorf tubes. The samples were centrifuged at 15,000 rpm for 5 min, and the supernatant was discarded. Subsequently, 2 mL of dH_2_O was added, vortexed, and the samples were centrifuged again at 15,000 rpm for 5 min. After discarding the liquid portions, the samples were treated with 2 mL of 90% acetone and vortexed. These vortexed samples were incubated at 80 °C for 15 min in an oven, then immediately centrifuged at 15,000 rpm for 5 min. Following centrifugation, supernatant was measured using a UV-Vis spectrophotometer against a 90% acetone blank. Absorbance values were recorded at 750 nm, 664 nm, 647 nm, 630 nm, and 470 nm, marking the end of the experiment [23].

Chlorophyll and carotenoid concentrations were calculated using the following formulas:

I. Chlorophyll-*a* = 11.93*A_664_ – 1.93*A_647_
II. Chlorophyll-*b* = 20.36*A_647_ – 5.50*A_664_
III. Total carotenoid = (1000*A_470_) – (2.77*Cl-*a*)/213

### Measurement of cell density

Samples collected from the cultures were placed into sedimentation tubes and fixed with Lugol’s solution. Cell counts were then performed using a Leica DM-IL inverted microscope at the Environmental Laboratory of the Biology Department [2, 55]. The cell counting procedure was conducted using the Utermöhl method, a well-established counting technique in the literature.

### Measurement of cell photosynthetic fluorescence

The optical density (OD) values of cultures treated with distinct electrical waveforms were measured using the AquaPen-C AP-C 100 device. Throughout the experiments, on days when distinct electrical waveforms were applied, samples were taken from the cultures in a sterile cabinet and dark-adapted for at least 10 min in a darkroom. The device was calibrated with TAP liquid medium, and OD_680_ nm, OD_720_ nm, and OJIP values were measured sequentially.

### Analysis of FTIR spectroscopy

This method, based on the principle of measuring the vibrational modes of chemical bonds through the absorption of infrared (IR) radiation, utilized the Bruker Vertex 70 V model FTIR spectrometer. Approximately 5 ± 1 mg of dried algal sample was placed onto the instrument’s ATR attachment. Biomolecules were identified by measuring their absorption results in the 4000–400 cm⁻¹ wavenumber region (MIR region) with a 4 cm⁻¹ resolution [36, 40].

### HPLC instrumentation

HPLC analysis was performed to determine the carotenoid profile [21]. The HPLC system comprised two pulse-free solvent pumps (one being a 3-channel DGU-20A5), an SPD-M20A photodiode array detector, and the LabSolutions System chromatography software (Shimadzu Corporation, Kyoto, Japan). For carotenoid separation, a Develosil^®^ 5 μm C30-UG 140 Å, LC Column (250 × 4.6 mm) with a C30-UG phase and 140 Å pore size (1 Ångström = one hundred-millionth of a centimeter) was used. The mobile phases had a flow rate of 0.6 mL/min and consisted of (A) methanol-acetonitrile-water (84:14:2, v/v/v) and (B) 100% methylene chloride. The initial gradient solvent system, starting at 100% A phase and 0% B phase, decreased to 95% A phase in 8 min, 75% A phase in 25 min, 72% A phase in 30 min, and 45% A phase in 50 min. It then returned to 100% A phase in 52 min and was maintained for 8 min. The extract injection volume was 10 µL, and peak responses were determined at 450 nm.

### Statistical analyses

Significant differences between samples treated with various electric waveforms and the control group were determined using Student’s t-test at a 5% significance level (*P* < 0.05), carried out with Microsoft Excel 2016. All experiments were designed as three independent series, with data representing mean values and standard errors.

## Results and discussion

### Effects of electrochemical treatment on *C. vulgaris*

**Fig. 2:**
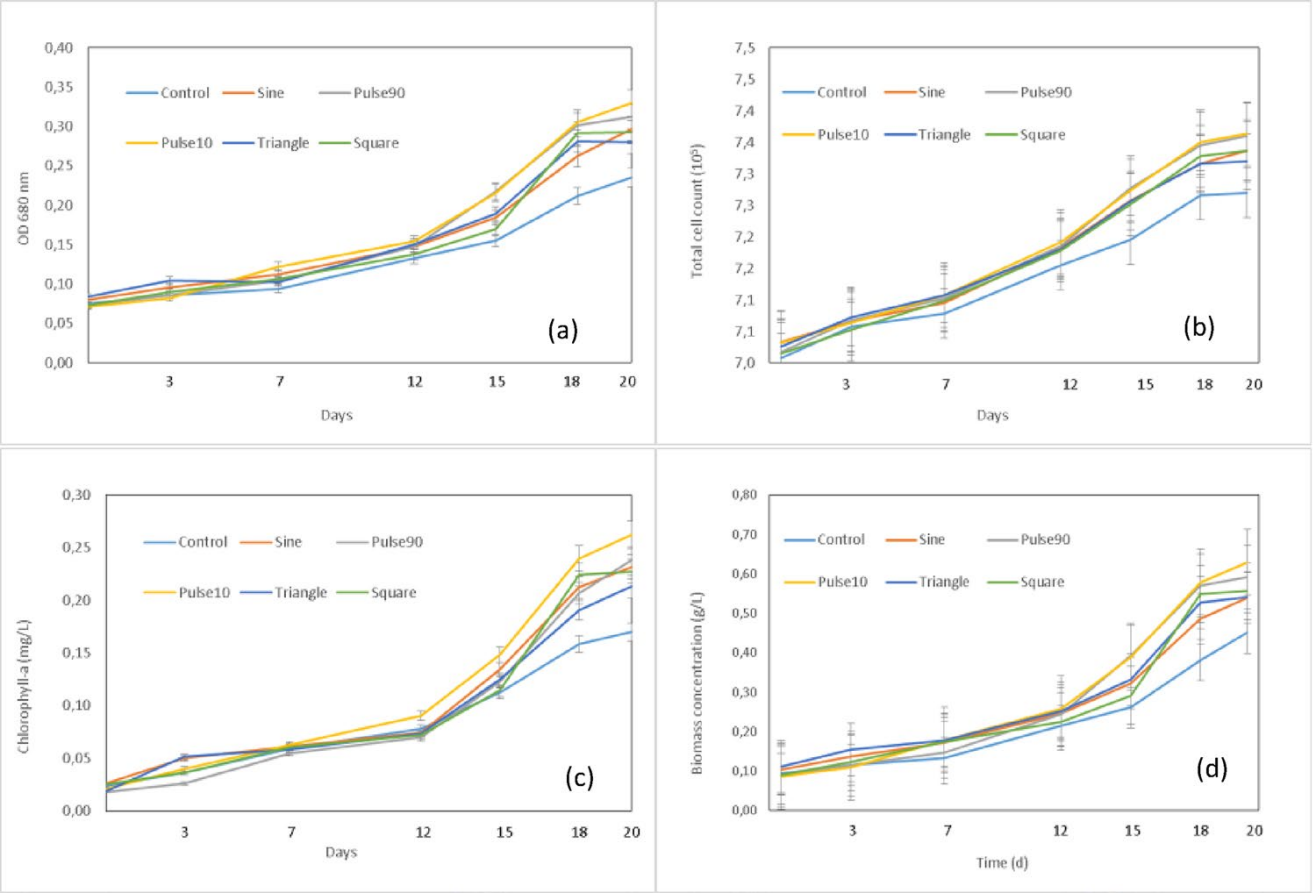
The effect of distinct alternating current waveforms on microalgae cultivated in TAP medium: **a **OD_680_ **b **Microalgal cell count **c **Microalgal biomass **d **Microalgal chlorophyll-*a* concentration

In the current study, the *C. vulgaris* strain was selected for its potential to support increased biomass production under various electrical waveform configurations. *C. vulgaris* cells at the end of their log growth phase were inoculated into sterilized TAP medium, starting at an OD_680_ of 0.2. The effects of electrochemical treatments were investigated daily using distinct electrical waveform configurations. This research chose four indicators to characterize the *C. vulgaris* cultivation process: optical density (OD), cell count, chlorophyll-*a* content, and dry weight. Applying various electrical waveforms revealed a significant shift in growth kinetics for the treated groups compared to the control, indicating a positive impact on microalgal growth performance. Similarly, electrical applications to date are known to enhance growth. The study by Kim et al. [25] reported that direct current increased growth by 1.2 times, and various alternating current applications, such as direct current electric and field applications, also stimulated growth. The experimental results are presented in Fig. 2. Figure 2a illustrates the increase in *C. vulgaris* OD_680_ following the application of various alternative currents throughout the cultivation period. The OD_680_ values of cultures exposed to distinct electric waveforms were significantly higher (*p* < 0.05) than those of the control group. Despite the limited changes observed throughout the growth phase, a subsequent series of events was observed at harvest time: Pulse-10 > Pulse-90 > Sine > Square > Triangle. Increases of 40%, 33%, 26%, 24%, and 19% were observed, respectively, compared to the control group. Under electrochemical treatment conditions with distinctt electric waveforms in TAP medium, the cell count of *C. vulgaris* was significantly higher (*p* < 0.05) than that of the control group. As shown in Fig. 2b, the same hierarchical order of effectiveness was maintained was determined to be Pulse-10 > Pulse-90 > Sine > Square > Triangle. Increases of 19%, 19%, 14%, 14%, and 11% were observed, respectively, compared to the control group. Concurrently, this research also evaluated the chlorophyll-*a* content produced under distinct electric waveforms. Changes in chlorophyll-*a* content were similar to those observed in OD_680_ as depicted in Fig. 2c. The chlorophyll-*a* content of *C. vulgaris* significantly increased (*p* < 0.05) by 54%, 40%, 36%, 34%, and 25%, respectively, compared to the control group.

Figure 2d displays the dry weight of *C. vulgaris* measured throughout the cultivation period. After cultivation, the dry weight of *C. vulgaris* in the Pulse-10 electrical treatment group reached 0.63 g/L, while Pulse-90 achieved 0.59 g/L, Square 0.56 g/L, and both Triangle and Sine groups were at 0.54 g/L. Compared to the control group, these values represent a statistically significant increase (*p* < 0.05) of 40%, 31%, 23%, 20%, and 20%, respectively. Microscopic observations during cultivation revealed no aggregation or disintegration of microalgal cells subjected to the distinct electric waveforms. This absence of adverse effects suggests that the short application duration (60s) and low intensity (7.30 J/second) likely contributed to this outcome. The literature features numerous studies on coagulation and extraction using high electric field applications [15, 32, 61]. These four indicators collectively demonstrate that the applied electric waveforms promoted the growth of *C. vulgaris*. Microscopic observations, which showed most microalgal cells in a state of division, align with the OD_680_ data and cell count results. This indicates that the electrochemical treatment stimulates cell division without causing deformation in the cells [26]. The observed positive correlation between chlorophyll content and microalgal growth further indicates that the distinctelectric waveforms positively influenced *C. vulgaris* cells, increasing both cell numbers and, consequently, chlorophyll-*a* content. These findings are consistent with similar studies in the literature [26, 44, 60].

### Photosynthetic effect of distinct electric waveforms on *C. vulgaris*

The primary source of information regarding processes within the photosynthetic chain is chlorophyll fluorescence, with the majority originating from Photosystem II (PSII) and its associated light-harvesting complexes. Chlorophyll fluorescence serves as a widely utilized methodology, offering fundamental insights into PSII function [13]. The intensity of this fluorescence signal largely depends on the state of the PSII reaction center. Photosystem I (PSI) contributes only minimally to the overall fluorescence signal, making it possible to attribute changes in total fluorescence to PSII [47]. The induction of chlorophyll-a fluorescence transients (OJIP), which is associated with the electron transfer process in PSII, reflects the photosynthetic activity of microalga. Parameters such as Fv/Fm, ABS/RC, TRo/RC, ETo/RC, and DIo/RC constitute key chlorophyll fluorescence metrics employed to assess the operational status and overall condition of the photosynthetic apparatus, specifically gauging the maximum quantum efficiency and stress levels of PSII [31, 37]. ETo/TRo quantifies the ratio of energy absorbed at the reaction center that is subsequently channeled into downstream electron capture reactions [30, 59]. An elevation in ETo/TRo values signifies the efficient operation of PSII and the robustness of electron transfer [65]. Furthermore, the observation of no substantial deviation in ETo/RC and TRo/RC values indicates that the mean electron transfer efficiency within PSII is maintained at a stable level (Yan et al., 2023). Fv/Fm changes can indicate the degree of treatment and photosynthetic characteristics of microalga. An increase in Fv/Fm values compared to the control group, driven by the effect of alternating current forms, suggests the activation of PSII reaction centers and an accelerated rate of photosynthesis, consequently leading to enhanced growth (Li et al., 2024) Table 1.

**Table 1:** Photosynthetic effect of distinctalternating current waveforms on microalgae cultivated in TAP medium

		0. Day	3. Day	7. Day	12. Day	15. Day	18. Day	20. Day
Control	Eto/RC	0.4260	0.4040	0.4255	0.5105	0.4555	0.4605	0.5090
	Eto/Tro	0.6025	0.6382	0.7032	0.7818	0.7579	0.7593	0.8151
	RC/ABS	0.9815	0.8825	0.7615	0.8540	0.8040	0.8250	0.8170
	Fv/Fm	0.6605	0.7170	0.7480	0.7655	0.8095	0.7355	0.7650
Sine	Eto/RC	0.4935	0.4295	0.4095	0.5650	0.4750	0.4935	0.5165
	Eto/Tro	0.7173	0.6769	0.7064	0.8389	0.7781	0.7840	0.8102
	RC/ABS	0.9470	0.8600	0.7775	0.8585	0.8030	0.8395	0.8375
	Fv/Fm	0.7265	0.7380	0.7485	0.7845	0.8450	0.7495	0.7615
Pulse-90	Eto/RC	0.4830	0.4015	0.4375	0.5945	0.5555	0.4910	0.5015
	Eto/Tro	0.6930	0.6409	0.7214	0.8415	0.8273	0.7769	0.7916
	RC/ABS	0.9645	0.8635	0.7735	0.8020	0.8650	0.8440	0.8375
	Fv/Fm	0.7225	0.7260	0.7565	0.7835	0.8890	0.7485	0.7565
Pulse-10	Eto/RC	0.5050	0.4215	0.4585	0.5345	0.5385	0.5445	0.5300
	Eto/Tro	0.7073	0.6717	0.7373	0.8105	0.8171	0.8085	0.7976
	RC/ABS	0.9765	0.8540	0.7625	0.8455	0.8560	0.8880	0.8990
	Fv/Fm	0.7310	0.7350	0.7640	0.7800	0.8425	0.7580	0.7390
Triangle	Eto/RC	0.4835	0.4300	0.4885	0.5375	0.5365	0.4875	0.5245
	Eto/Tro	0.6982	0.6620	0.7413	0.8071	0.8197	0.7757	0.7461
	RC/ABS	0.9500	0.8730	0.7860	0.8600	0.8480	0.8440	0.8900
	Fv/Fm	0.7285	0.7440	0.7650	0.7740	0.9150	0.7445	0.7010
Square	Eto/RC	0.4575	0.4225	0.4560	0.5705	0.5450	0.5320	0.5370
	Eto/Tro	0.6718	0.6520	0.7065	0.8310	0.8295	0.8067	0.8081
	RC/ABS	0.9715	0.8870	0.7800	0.8750	0.8415	0.8685	0.8900
	Fv/Fm	0.7010	0.7305	0.7455	0.7845	0.9070	0.7590	0.7470

The chlorophyll fluorescence parameters characterizing photosynthetic activity demonstrate that the application of distinct waveform types enhances the photosynthetic efficiency in *C. vulgaris*. By day 3 of the experiment, a rapid increase in Fv/Fm to values between 0.71 and 0.73 surpassed the control group’s values. Notably, continuous periodic energization on day 12 further enhanced Chlorella’s photosynthetic performance. However, following the 15th day, a slight decrease was observed in ETo/RC, ETo/TRo, and Fv/Fm toward the end of the incubation period. This decline is likely attributed to the increased cell density, which can lead to reduced light penetration and decreased photosynthetic efficiency (Li et al., 2024). These results are consistent with existing literature on electric field applications, further demonstrating the growth-promoting properties of distinctt electric waveforms on algae. Qualitatively, these applications appear to facilitate energy capture by increasing the electrochemical response and relative photosynthetic electron transfer rate within algal cells. They polarize ionic charges within the cell and accelerate electron transfer, which in turn promotes cell permeabilization and facilitates nutrient uptake, ultimately leading to increased biomass [7, 26, 42, 49].

### Carotenoid effect of distinct electric waveforms on *C. vulgaris*

**Fig. 3:**
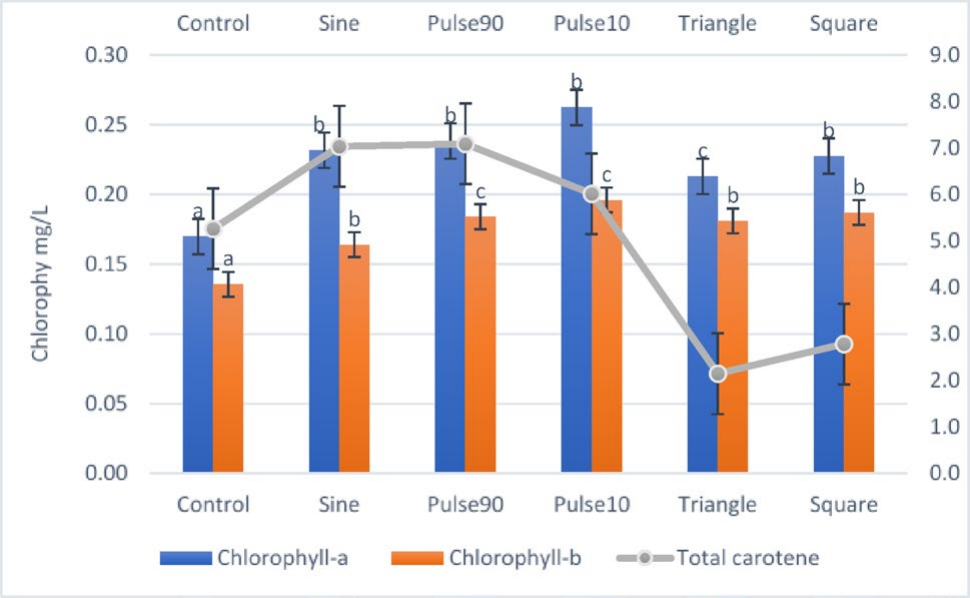
Impact of various alternating current waveforms on chlorophyll-*a* concentration and total carotene content in microalgae cultured in TAP Medium

**Fig. 4:**
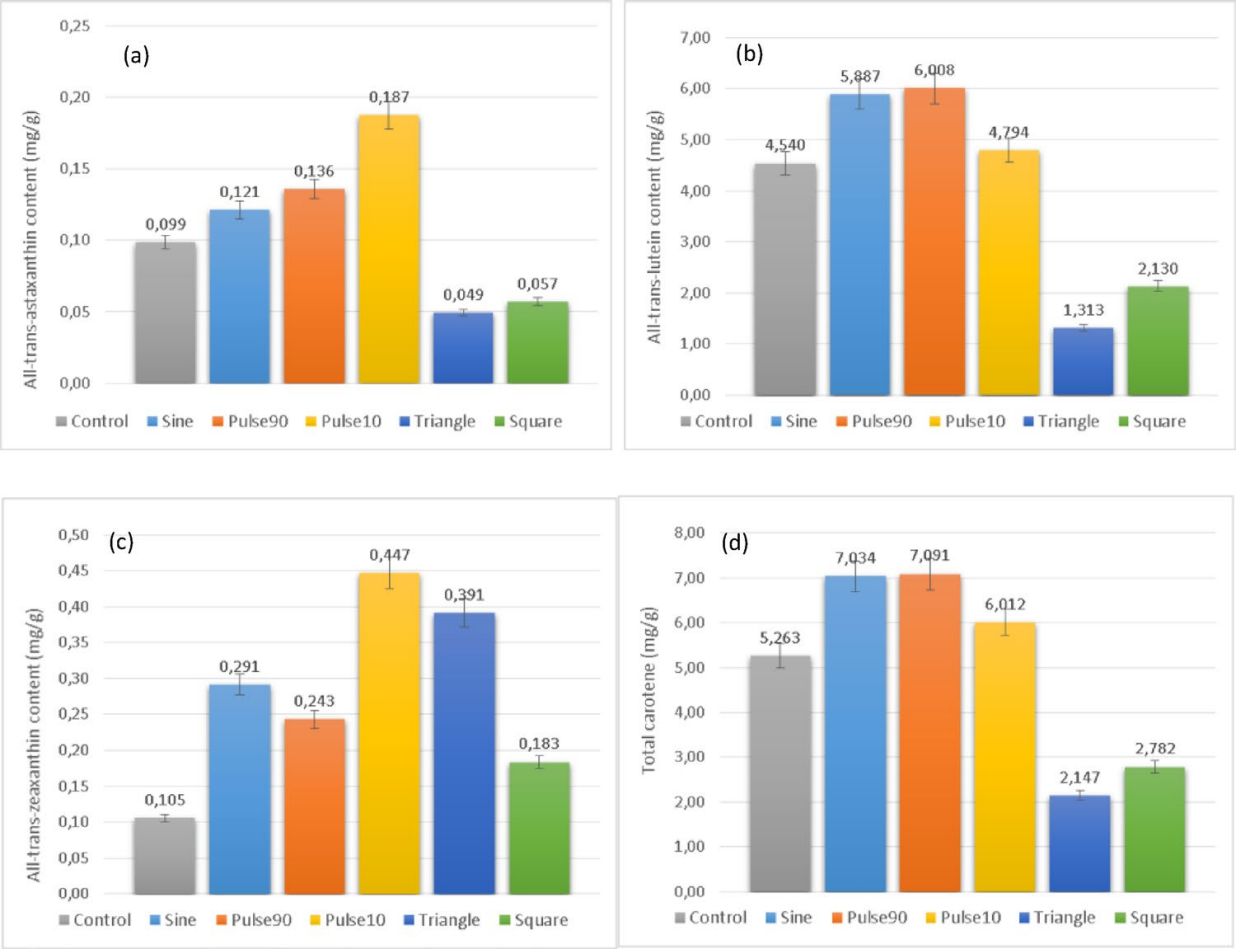
The effect of distinctt alternating current waveforms on microalgae cultivated in TAP medium: **a** *All-trans*-astaxanthin **b** *All-trans*-lutein **c ***All-trans*-zeaxanthin **d ***All-trans*-β-Carotene

Chlorophyll constitutes approximately 2% of *C. vulgaris*’s dry weight. Beyond this, carotenoids, which belong to the secondary metabolites group, are also present. Beta-carotenes, astaxanthin, canthaxanthin, and lutein are among the primary pigments identified [45]. In this study, the observed increase in carotenoid levels indicates the algae’s tolerance response. Carotenoids protect algae by quenching high amounts of energy during excitation and releasing it as heat [54]. The carotenoids found in microalga are essentially beta-carotenes, associated with lipids located in chloroplasts, and with chlorophyll and thylakoids within the chloroplasts themselves. Figure 3 illustrates the total carotenoid content of *C. vulgaris* treated with distinct electrical waveforms. Compared to the control group, the Pulse-90 waveform showed a 35% increase, the Sine waveform a 34% increase, and the Pulse-10 waveform a 14% increase in total carotenoid amounts. Conversely, a 50% reduction was observed with both the Triangle and Square waveforms. In the current study, total carotenoid content ranged from 2.47 to 7.09 mg/g, which aligns with existing literature. For instance Paliwal et al.,(2016) reported total carotenoid levels between 0.23 and 7.2 mg/g across 57 species belonging to the Chlorophyta and Cyanophyta groups. Furthermore, the potential carotenoid content of *C. vulgaris* has been reported to vary significantly depending on distinct culture media and conditions, with ranges such as: Chlorophyll-*a* (0.25–9.63 mg/g), Chlorophyll-*b* (0.072–5.77 mg/g), Lutein (0.052–3.83 mg/g), β-Carotene (0.007–12 mg/g), Astaxanthin (up to 550 mg/g), and Cantaxanthin (up to 362 mg/g) [48]. All electrical waveforms applied to *C. vulgaris* altered its carotenoid composition, specifically impacting All-trans-astaxanthin, All-trans-lutein, All-trans-zeaxanthin, All-trans-β-cryptoxanthin, and All-trans-β-Carotene levels compared to the control group. The most pronounced changes were observed with the Pulsed electric waveforms. Notably, the Pulse-10 waveform led to significant increases in All-trans-astaxanthin and All-trans-zeaxanthin, while the Pulse-90 waveform resulted in substantial elevations of All-trans-lutein and All-trans-β-Carotene (Supplemantary Figs. 6–11). Samples exposed to the Sine electrical waveform exhibited increases of 23%, 30%, 176%, and 24%, respectively. Regarding the specific carotenoid composition (All-trans-astaxanthin, All-trans-lutein, All-trans-zeaxanthin, and All-trans-β-Carotene): Pulse-90 electrical waveform application resulted in increases of 38%, 32%, 131%, and 66%, respectively. Pulse-10 electrical waveform application led to even higher increases of 90%, 6%, 324%, and 37%, respectively. Conversely, samples treated with Triangle and Square electrical waveforms showed a similar fifty-percent reduction in both All-trans-astaxanthin and All-trans-β-Carotene levels. However, All-trans-zeaxanthin content increased by 271% with the Triangle waveform and 74% with the Square waveform (Fig. 4).

r

### Macromoleculer effect of distinct electric waveforms on *C. vulgaris*

Microalgae are attractive as potential sources for feed and food applications [8]. We analyzed the principal macromolecules of biomass obtained by exposing it to distinct electrical waveforms using FTIR analysis. FTIR spectroscopy is widely used to provide information about a range of vibrational functional groups that characterize biomolecules. It has been shown to be a highly effective method for evaluating the functional groups in algal biomass, both in terms of their presence and subsequent changes [36, 40]. This study utilized FTIR spectroscopy to characterize changes in the chemical functional groups of *C. vulgaris* cells treated with distinct electrical waveforms. While the peak shifts for *C. vulgaris* treated with various electrical waveforms were similar to those in the control group, the absorption peak values significantly increased (Supplemantary Fig. 5). These data indicates that distinct electrical waveforms not only support biomass increase but also macromolecule production. The presence of lipids in the samples was evident in the spectral range between 3000 cm⁻¹ and 2800 cm⁻¹, attributed to the symmetric and asymmetric stretching vibrations of -CH₂- groups [53]. These -CH₂- groups are an integral part of the lipid backbone structure, and in the current study, they were represented by a peak at 2925 cm⁻¹. *C. vulgaris* protein spectra were characterized by the presence of strong peaks at 1628 cm⁻¹ (amide I) and 1536 cm⁻¹ (amide II) [53]. These primary bands result from the C = O stretching vibration and a combination of N-H and C-N stretching vibrations within amide complexes. Carbohydrates exhibited weak to medium bands in the 1150–839 cm⁻¹ wavenumber range [20, 41, 50, 53]. In the current study, C-O stretching vibrations were represented by a peak at 1036 cm⁻¹. The intensities of all bands representing macromolecules in this study were determined to be in the order of Pulse-90 > Triangle > Sine > Pulse-10 > Square > Control. These findings indicate an increase in macromolecules in the treated groups compared to the control. He et al., [17] reported that stimulating *Scenedesmus obliquus* with a 1 A current for 7 days increased lipid yield by 77% compared to the control. Similarly, Silva et al., [51] reported a significant enhancement in lipid accumulation in *D. salina* with low electrical stimulation. Achieving higher production with lower energy input (mA currents) could potentially make industrial-scale application more feasible. This study’s findings align with existing electrochemical studies in the literature that aim to enhance macromolecule production [11,26,33,17].

### Analysis of waveform parameters and energy dynamics

All waveforms were applied to Chlorella vulgaris samples at a constant frequency of 10 kHz and a peak-to-peak voltage of 10 V to ensure a controlled comparative baseline. Despite identical frequency and voltage settings, the biological response varied drastically according to the wave geometry. As illustrated in Supplemantary Figs. 6–11, the levels of All-trans-astaxanthin, All-trans-lutein, All-trans-zeaxanthin, and All-trans-β-Carotene showed substantial increases in the Pulse-10, Pulse-90, and Sine groups compared to the control. Specifically, these waveforms triggered proportional carotenoid yield increases of 324%, 176%, and 131%, respectively. A comprehensive evaluation of RMS voltage, instantaneous power, and total energy delivered over 60 s reveals that power magnitude alone is not the determining factor for yield. For instance, the Square wave, which caused a 50% yield loss, delivered the second-highest power (7.31 W) and energy (438.6 J). In contrast, Pulse-90 delivered the highest power (13.16 W) and energy (789.6 J) but resulted in a massive yield increase. This suggests that the algae’s response is not negatively affected by high power itself, but rather by the manner in which the power is delivered (Table 1).

### Gradual power modulation of electrostimulation waveforms for optimized algal growth

Fast Fourier Transform (FFT) spectral analysis, conducted via digital oscilloscope, revealed that although all waveforms were driven at a fundamental frequency of 10 kHz, only the Pulse-10 and Pulse-90 configurations exhibited a significant secondary harmonic component at 20 kHz. The Pulse-10 dynamics were characterized by a stabilized power oscillation, fluctuating moderately between 4 and 8 dB at the primary frequency (10 kHz) and 0–5 dB at the secondary harmonic (20 kHz). This rhythmic, periodic modulation—analogous to a controlled “wave-like” stimulation—appears to optimize metabolic momentum within the microalgae, fostering enhanced growth. Similarly, the Sine wave, distinguished by its continuous transitions and lack of abrupt harmonics, achieved the highest yield among non-pulsed waveforms due to its inherent signal fluidity.

In contrast, the “Shock Factor” induced by the sharp geometries of the Square and Triangle waves led to a substantial 50% reduction in yield. The instantaneous and repetitive voltage transitions characteristic of the square wave likely induced metabolic arrest. Quantitatively, since the decibel (dB) scale is logarithmic, the 13.2 dB peak recorded in the square wave signifies a 20-fold increase in power intensity compared to baseline levels. These findings suggest that while total power delivery is a critical factor, the rate of change (dV/dt) is the primary determinant of physiological response; gradual transitions facilitate metabolic uptake, whereas abrupt, high-intensity transitions trigger detrimental physiological stress and systemic inhibition (Table 2).

**Table 2.** Electric parameters of distinct alternating current waveforms on microalgae cultivated in TAP medium

	RMS voltage	Average power	60 second total power	Sub frequency harmonics power and power interval	
Control				10 Khz	20 Khz
Sine	3,54 Volt	1,83 W	110 J	Exists 10,8dB	Absent
Pulse-90	9,49 Volt	13,2W	790 J	Exists between 3-9 dB changes	Exists between 3-8 dB changes
Pulse-10	3,16 Volt	1,46 W	87,7 J	Exists between 4-8 dB changes	Exists between 0-5 dB changes
Triangle	2,89 Volt	1,22W	73,2 J	Exists 9,6dB	Absent
Square	7,07 Volt	7,31 W	439 J	Exists 13,1 dB	Absent

The application of FFT analysis renders such raw electrophysiological data biologically meaningful. By converting time-domain signals into a frequency-domain power spectral density, even faint resonance peaks can be precisely quantified. This methodology is consistent with advanced spectroscopic techniques; for instance, in the characterization of bioactive compounds in Indonesian Red Algae, FFT serves as the fundamental computational engine for FTIR spectroscopy [56]. By converting complex interferogram data into readable spectra, FFT allows for the precise identification of chemical signatures—such as sulfated polysaccharides—that correlate with therapeutic potential. In a similar vein, our dB-based power analysis provides a sensitive metric for evaluating thermal-acoustic conversion efficiency, revealing the extent to which electrical energy is utilized for metabolic processes versus dissipated as heat at the single-cell level [43].

### PCA integration and biochemical correlations

The clustering observed in the Principal Component Analysis (PCA) plots provides a robust biochemical foundation for these findings. The spatial positioning of the groups is in perfect harmony with the yield data. Efficient Groups (Pulse-90, Pulse-10, Sine) succeeded. In the PCA plots (specifically Fv/Fm and RC/ABS), these three groups shift significantly away from the control toward the positive PC1 axis (the right side). Pulse-10 & Pulse-90 effect, these forms deliver electrical stimuli in “bursts.” *C. vulgaris* responds to these rapid yet interval-based stimulations by exponentially increasing photoprotective carotenoids (e.g., Lutein, Zeaxanthin) to absorb the energy and prevent electro-photo damage. Sine effect, the smooth gradient of the Sine wave places the reaction centers into a more efficient “active standby” mode. This is reflected in its distinct position in the RC/ABS plot, indicating a synergy that supports steady carotenoid accumulation. Why inefficient groups (Triangle & Square) failed, the 50% yield loss in these groups is explained by their outlier positions in the PCA data. The triangle paradox, in the ETo/Tro and Fv/Fm plots, the Triangle wave (purple dot) appears as an extreme outlier at the far ends of the axes. The linear but constant rise and fall of electrical intensity does not allow for sufficient “rest periods,” leading to chronic stress. This stress suppresses the efficiency of the photosynthetic apparatus (PSII) and blocks carotenoid synthesis pathways. The square wave Impact, the instantaneous jump to maximum power acts as a repetitive shock, rendering the Reaction Centers (RC) inefficient, as evidenced in the RC/ABS plot. Instead of synthesizing carotenoids, the cell enters a “survival mode,” diverting its metabolic energy away from secondary metabolite synthesis and toward cellular repair mechanisms (Supplemantary Fig. 12a-d).

## Conclusions

Results obtained from distinct electrical waveform processing methods have contributed to intensive global electro-technology research conducted in recent years. In this study, we investigated the effects of various electrical waveforms on the growth, photosynthetic activity, macromolecular and carotenoid composition of *C. vulgaris*. Our findings demonstrate that specific AC geometries, particularly the Pulse-10 and Pulse-90 waveforms, effectively accelerate microalgal proliferation through electrochemical stimulation. We observed statistically significant enhancements in optical density, cell density, chlorophyll-a concentration, and biomass dry weight. Notably, the Pulse-10 waveform consistently yielded the most pronounced improvements across all growth parameters, suggesting that tailored periodic electrical stimulation serves as a potent growth promoter. These results are congruent with existing literature regarding the application of electric fields in microalgal biotechnology. Furthermore, photosynthetic performance analysis revealed that these waveforms enhanced the quantum efficiency of Photosystem II (PSII), as evidenced by the marked increase in F*v*/F*m* values, indicating a more robust and active photosynthetic apparatus under specific electrical modulation.

These results are directly related to increased photosynthesis rates and growth improvements. Furthermore, it was found that electrochemical treatment positively affected the macromolecular composition and increased the total content of lipids, proteins, and carbohydrates, which are crucial for biomass quality. In particular, the carotenoid profile was significantly altered by distinct waveforms; Pulse-10 and Pulse-90 led to a significant increase in beneficial compounds such as astaxanthin, lutein, and β-carotene, which are vital for both the treatment tolerance of algae and their value in food and feed applications. This has enabled efforts to increase the production of valuable compounds such as carotenoids, which are used in various sectors including health (medicine and pharmaceuticals) and food.

## Supplementary Information

Below is the link to the electronic supplementary material.

Supplementary Material 1

## Data Availability

No datasets were generated or analysed during the current study.
