# Supplementary material for: Electrostimulation waveform optimization for enhancing biomass and macromolecule production in Chlorella vulgaris
Source: Bioprocess Biosyst Eng. 2026 Jun 10;49(6):1707–17. doi: 10.1007/s00449-026-03355-1 (PMC13328133; doi:10.1007/s00449-026-03355-1)
Supplement: Supplementary file 1 — Supplementary Material 1 [file 449_2026_3355_MOESM1_ESM.docx]

- Control
- Square
- Pulse-10
- Pulse-90
- Sine
- Triangle

**Figure 5.** Effect of different alternating current waveforms on microalgae cultivated in TAP medium: FTIR spectrum.

**Figure 6.** Effect of triangular alternating current waveform on cultivated microalgae; HPLC chromatogram of carotenoids extracted from *Chlorella vulgaris.*

**Figure 7.** Effect of sinüs alternating current waveform on cultivated microalgae; HPLC chromatogram of carotenoids extracted from *Chlorella vulgaris.*

**Figure 8.** Effect of Pulse-90 alternating current waveform on cultivated microalgae; HPLC chromatogram of carotenoids extracted from *Chlorella vulgaris.*

**Figure 9.** Effect of Pulse-10 alternating current waveform on cultivated microalgae; HPLC chromatogram of carotenoids extracted from *Chlorella vulgaris.*

**Figure 10.** Effect of square alternating current waveform on cultivated microalgae; HPLC chromatogram of carotenoids extracted from *Chlorella vulgaris.*

**Figure 11.** Effect of control cultivated microalgae; HPLC chromatogram of carotenoids extracted from *Chlorella vulgaris.*


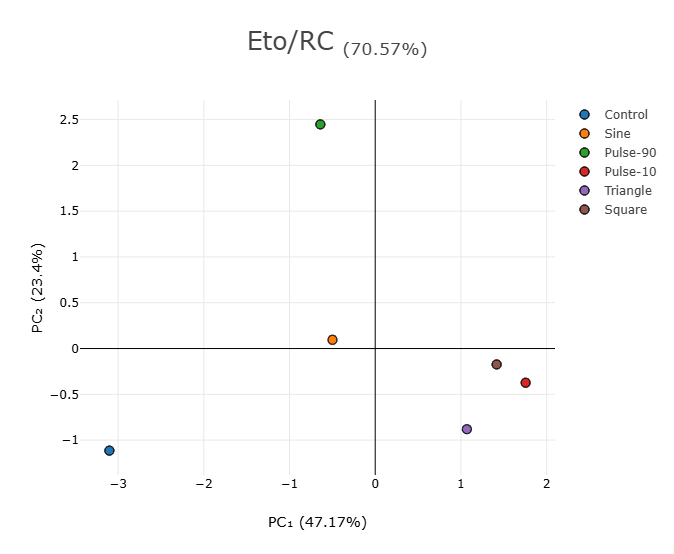


**Figure 12a**.PC analysis of Eto/RC


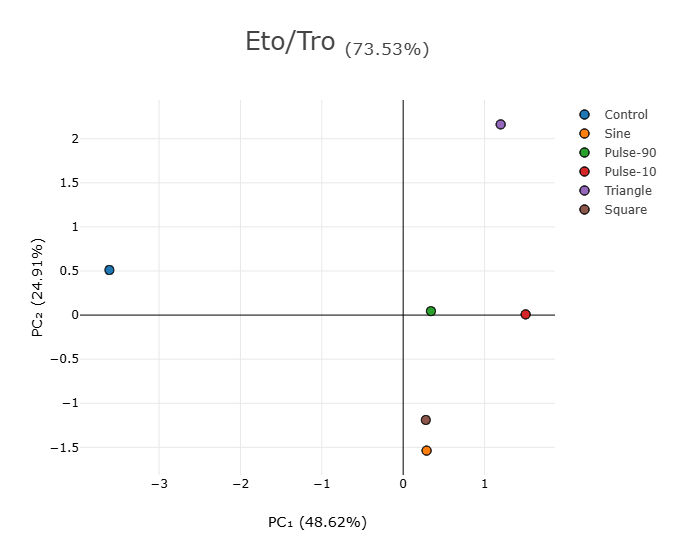


**Figure 12b**. PC analysis of Eto/RC


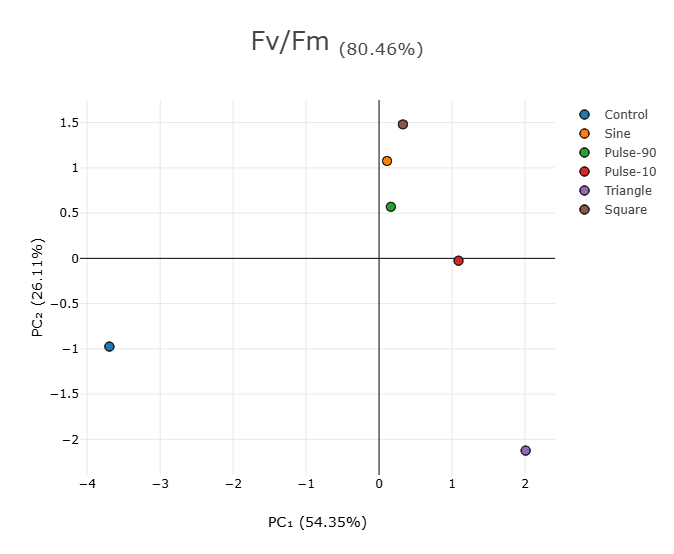


**Figure 12c**. PC analysis of Fv/Fm


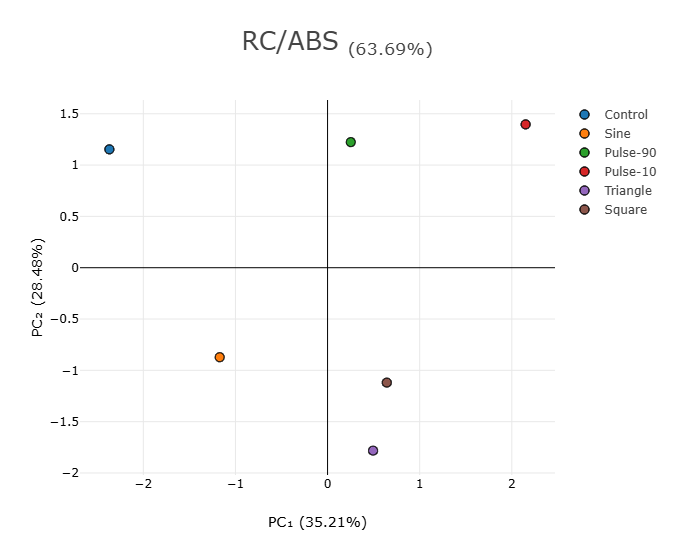


**Figure 12d**. PC analysis of RC/ABS
